# Cortical Thinning and Ventricle Enlargement in Neuromyelitis Optica Spectrum Disorders

**DOI:** 10.3389/fneur.2020.00872

**Published:** 2020-08-27

**Authors:** De-Cai Tian, Yuwen Xiu, Xinli Wang, Kaibin Shi, Moli Fan, Ting Li, Huining Li, Lei Su, Yuetao Ma, Wangshu Xu, Tian Song, Yaou Liu, Fu-Dong Shi, Xinghu Zhang

**Affiliations:** ^1^Center for Neuroinflammation, Beijing Tiantan Hospital, Capital Medical University, Beijing, China; ^2^China National Clinical Research Center for Neurological Diseases, Beijing, China; ^3^Department of Neurology, Tianjin Neurological Institute, Tianjin Medical University General Hospital, Tianjin, China; ^4^Department of Radiology, Beijing Tiantan Hospital, Capital Medical University, Beijing, China

**Keywords:** NMOSD, MS, degeneration, AQP-4, CSF–brain barriers

## Abstract

**Background:** In neuromyelitis optica spectrum disorders (NMOSDs), inflammation is not the sole driver of accumulation of disability; neurodegeneration is another important pathological process. We aim to explore different patterns of cortical atrophy and ventricular enlargement in NMOSD.

**Methods:** We retrospectively analyzed a cohort of 230 subjects, comprising 55 healthy controls (HCs), 85 multiple sclerosis (MS), and 90 NMOSD patients from Tianjin Medical University General Hospital and Beijing Tiantan Hospital. Different compartments of the brain (total gray, cortex, subcortex gray, and ventricle volume) were evaluated with the FreeSurfer. Multiple linear regressions were adopted to explore associations between cortex volume and predict factors.

**Results:** Compared with HCs, NMOSD, and MS displayed an enlarged lateral and third ventricle (*p* < 0.001), whereas expansion of the fourth ventricle was observed in MS rather than NMOSD (*p* = 0.321). MS and NMOSD patients exhibited cortical thinning in comparison with HCs. However, pronounced cortical atrophy were only significant in pre-cuneus, parahippocampal, and lateral occipital lobe between MS and NMOSD. Patients with NMOSD had decreased local gyrification index in orbitofrontal and pre-cuneus lobe, and reduced pial surface area. Linear regression analysis revealed cortex volume were predicated by advanced age (standardized β = −0.404, *p* = 0.001) as well as prolonged disease history (standardized β = −0.311, *p* = 0.006).

**Conclusion:** NMOSD exhibited global cortex atrophy with enlarged lateral and third ventricles. Moreover, cortex volume is associated with age and disease duration.

## Introduction

Multiple sclerosis (MS) and neuromyelitis optica spectrum disorders (NMOSDs) are autoimmune disorders of the central nervous system (CNS) with inflammation-mediated damage to oligodendrocyte and astrocytes (1). Inflammation and neurodegeneration co-exist as two main pathogenic components in both disease processes (2). In MS, clinical observations and pathological evidence show cortical neuronal loss does not directly correlate with white matter lesion accumulation, suggesting degeneration is a partially independent pathological process (3–6). Correspondingly, mild cortical thinning has also been described in normal-appearing brain tissue in MRI of patients with NMOSD (7). It is still unclear if such degeneration is an independent process or secondary to lesions in NMOSD.

Brain atrophy, ventricular enlargement, and widening of cortical sulci and gyri are even evident in conventional brain MRI in the large majority of MS patients (8). Ventricular enlargement, especially pronounced in the third ventricle, has been implicated as an early sign of progression, owing to deep gray matter involvement and periventricular tissue loss in MS. The distribution of lesions is more likely adjacent to lateral ventricle in MS. For NMOSD, lesions along the ependyma, a tissue type of high AQP-4 expression, especially around the third and fourth ventricles are distinctive. Furthermore, a significantly higher ratio of the third to fourth ventricular volume is prominent in NMOSD patients compared with MS and healthy controls (9).

Such distinct patterns of ventricular enlargement and cortical atrophy may indicate unique underlying pathological originations in both disease types. Similar to the blood–brain barrier, the blood–cerebrospinal fluid barrier (BCSFB) selectively permits blood-borne substances into the CNS (10). Cerebrospinal fluid (CSF) circulates through the ventricles and over the surface of the brain; CSF-mediated immune-pathological processes could thus be responsible for those tissue changes directly in contact with the CSF (such as periventricular regions and cortex). Recently, Jehna et al. reported that increased periventricular lesion burden was correlated with cortical thinning in patients with MS, supporting the notion that common CSF-mediated factors may play a role in the accumulation of damage to gray and white matter (11). Such implications are far reaching in the context of NMOSD. The BCSFB represents a potentially unique route for pathogenic NMO-IgG to enter the CSF and access AQP-4-expressing targets in those regions (12). The inflammatory sequelae of NMO-IgG binding to AQP-4 leads to the occurrence of ventriculitis and leptomeningitis in the histopathology of NMO (12). Loss of AQP-4 immunoreactivity was observed in cortical layer I and choroid plexus epithelium in NMO tissue, which is associated with cognitive impairment and a corresponding loss of neurons in cortical layer II (13). Concomitant to these pathological findings, brain atrophy and hydrocephalus were observed in NMOSD patients (14, 15).

Based on NMO IgG-induced pathological alterations at the ependymal CSF–brain and choroid plexus blood–CSF barriers, here we investigate evidence for different patterns of ventricular enlargement in a large cohort of patients with MS and NMOSD, and healthy controls. Brain components, at both global and regional levels, can be quantitated using surface-based methods for measuring cortical thickness, surface area, and folding. We also explored whether gray matter atrophy in NMOSD is a more diffuse “global” process or develops, instead, according to distinct anatomical patterns.

## Subjects and Methods

### Subjects

We retrospectively analyzed data of a cohort of 85 relapsing–remitting MS patients, 90 NMOSD patients, and 55 healthy controls, who completed comprehensive MRI and clinical examination from Tianjin Medical University General Hospital and Beijing Tiantan Hospital. Several independent image findings were published previously based on the current cohort (16–19). All MS patients were diagnosed according to 2017 MacDonald revised criteria (20), whereas NMOSD patients were based on the 2015 Wingerchuk criteria (1).

The NMOSD group had an average disease duration of 3 (1.8–6) years and a higher annualized relapse rate [1 (0.5–2.8)]. NMOSD patients were relatively older than MS (45.3 ± 14.3 vs. 37.3 ± 12, *p* < 0.01), although there was no difference in age (years) and disease duration [3 (1–6) years] in MS patients with NMOSD. The annual recurrence rate [0.8 (0.5–1.6)] and degree of disability measured by EDSS 2 (1–3) were lower in MS (Table 1). The study was conducted following the declaration of Helsinki. The local ethics committee had approved the study, and each participant had provided written informed consent.

**Table 1:** Demographic, clinical, and MRI measures.

**Variable**	**HC (*n* = 55)**	**MS (*n* = 85)**	**NMOSD (*n* = 90)**
**DEMOGRAPHIC AND CLINICAL INFORMATION**			
Female, *n* (%)	43 (78)	62 (73)	76 (84)
Age (MD ± SD), years	46.1 ± 12.9	37.3 ± 12	45.3 ± 14.3^##^
Duration of disease (MD, IQR), years	n/a	3 (1–6)	3 (1.8–6)
Annualized relapse rate (MD, IQR)	n/a	0.8 (0.5–1.6)	1 (0.5–2.8)^##^
EDSS (MD, IQR)	n/a	2 (1–3)	3 (2–4)^##^
AQP-4 Ab positivity, *n* (%)	n/a	n/a	59 (65.8%)
DMT treatment, *n* (%)	n/a	6 (7)	13 (14)
**BRAIN SEGMENT VOLUME**			
Total gray volume (cm^3^) (MD ± SD)	627.5 ± 53.9	601.7 ± 60.7^**^	591.6 ± 47.6^**^
Cortex volume (cm^3^) (MD ± SD)	464.2 ± 43.6	447.9 ± 47.3^*^	437.6 ± 38.1^**^
Sub-cortex gray volume (cm^3^) (MD ± SD)	57.38 ± 4.19	50.85 ± 7.14^**^	53.76 ± 4.84^**^^#^
Cerebral white matter volume (cm^3^) (MD ± SD)	452.8 ± 40.6	415.9 ± 59.7^**^	429.7 ± 44.7^**^
**VENTRICLE VOLUME**			
Lateral ventricle volume (cm^3^) (MD ± SD)	14.5 ± 7.6	23.6 ± 13.4^**^	18.1 ± 7.9^*^^##^
Inf-Lat-Vent volume (cm^3^) (MD ± SD)	0.63 ± 0.3	1.46 ± 1.3^**^	0.95 ± 0.5^**^^##^
3rd-Ventricle volume (cm^3^) (MD ± SD)	1.03 ± 0.3	1.72 ± 0.8^**^	1.3 ± 0.4^**^^##^
4th-Ventricle volume (cm^3^) (MD ± SD)	1.54 ± 0.4	1.76 ± 0.6^*^	1.53 ± 0.4^##^

*HC, healthy controls; MS, multiple sclerosis; NMOSD, neuromyelitis optica spectrum disorder; Inf-Lat-Vent, temporal horn of the lateral ventricle; EDSS, Expanded Disability Status Scale; MD, median; SD, standard deviation; IQR, interquartile range; DMT, disease-modifying therapy, including betaseron, teriflunomide, and rituximab*.

* *p < 0.05, compared with healthy controls*;

** *p < 0.01, compared with healthy controls*;

# *p < 0.05, compared with RRMS*;

## *p < 0.01, compared with RRMS*.

### Methods

#### MRI Data Acquisition

MR images were acquired using a MAGNETOM GE 3.0 Tesla MR scanner with an eight-channel head coil (Discovery MR750; General Electric, Milwaukee, WI, USA) at Tianjin Medical University General Hospital and Tiantan Hospital. The major parameters of sagittal 3D T1W structural images were as follows: TR/TE = 8.2/3.2 ms; FOV (field of view) = 256 × 256 mm; matrix size = 256 × 256; section thickness = 1 mm; no gap; 188 sagittal sections. The T2-weighted images were acquired by using a fast spin-echo sequence with the following parameters: TR/TE = 6,816/93 ms; FOV = 240 × 240 mm; section thickness = 6 mm; section gap = 1.5 mm; 20 axial sections. Axial T2-FLAIR (fluid-attenuated inversion recovery) were acquired with the following parameters: TR/TE/TI = 8,400/151.02/2,100 ms; FOV = 240 × 240 mm; matrix size = 512 × 512; section thickness = 6 mm; section gap = 1.5 mm.

#### MRI Data Processing: Brain Lesion Probability Map

Brain lesion probability map of MS and NMOSD patients were generated according to the following steps using the imaging tool of the Functional MRI of the Brain's Software Library (FSL, http://www.fmrib.ox.ac.uk/fsl). Mask of lesions were drawn on the T2 sequence by two experienced independent radiologists using MRIcron software, the rater identified the lesion in each slice and also referenced T2-FLAIR sequence and T1 sequence, then saved the lesion map as a binary mask. The two raters (Y.X. and X.W.) were blinded for the respective diagnosis, and the inter-rater reliability was 0.92. Subsequently, individual spatial T2WI images of patients with brain lesions of NMOSD and MS were first registered to T1WI images of individual space using the FRIRT-FMRIB's Linear Image Registration Tool, then individual space T1WI images were registered to the 2 mm T1WI template of the Montreal Neurological Institute (MNI152), and the T2WI image and the lesion mask of the individual space were finally written into the MNI space using the registration parameters to obtain the T2WI image and the lesion of the MNI standard space mask.

#### Volumetric MRI Parameters: Source-Based Morphometry

Normalized volumes of target brain compartments (total gray volume, cortex volume, subcortical gray volume, and ventricle volume) were quantified with FreeSurfer image analysis suite (v5.3.0), which is documented and freely available for download online (http://surfer.nmr.mgh.harvard.edu/). The pipeline of this software includes the removal of non-brain tissue, automated Talairach transformation, segmentation of gray matter (GM) and white matter (WM), intensity normalization, identification of WM and GM boundaries, and accurate correction of topological defects. Ventricle volume, cortical volume, thickness, pial area, and local gyrification index (LGI) maps of each subject were registered to the FreeSurfer “fsaverage” template and smoothened with a full-width at half-maximum of 10 mm before further statistics. Technical details of those parameters are described in previous publications (21, 22).

### Statistics

Statistical analyses of demographic, clinical, and MRI variables were performed in SPSS 22 (Chicago, IL, USA). Normality of the variables were assessed by Kolmogorov–Smirnov tests and histogram. Volumes of ventricles that did not fit normal distribution were log 10-transformed to meet the assumptions of inferential statistics. Results were Bonferroni corrected if applicable. *P*-values < 0.05 were considered statistically significant. One-way ANOVA was used between three groups, comparing different patterns of ventricle changes. Each subject's MRI data were resampled into a common space with a smoothing factor of 10 mm full-width at half-maximum. Then, one-way ANCOVA was adopted for cortical thickness comparisons between groups using age and gender as covariates. To explore possible associations with patterns of cortical thickness in the patients with NMOSD, partial correlations were computed between GM (total, cortical, and subcortical) volume and age, history, AQP-4 positivity, annualized relapse rate, and the length of myelitis. Moreover, to explore the factors influencing cortical thickness patterns among groups, stepwise linear regression analyses were performed using cortical GM volume, with dependent variables age, history, AQP-4 positivity, annualized relapse rate, and the length of myelitis as candidate predictors.

## Results

### Distribution of Brain Lesions in NMOSD and MS

**Figure 1:**
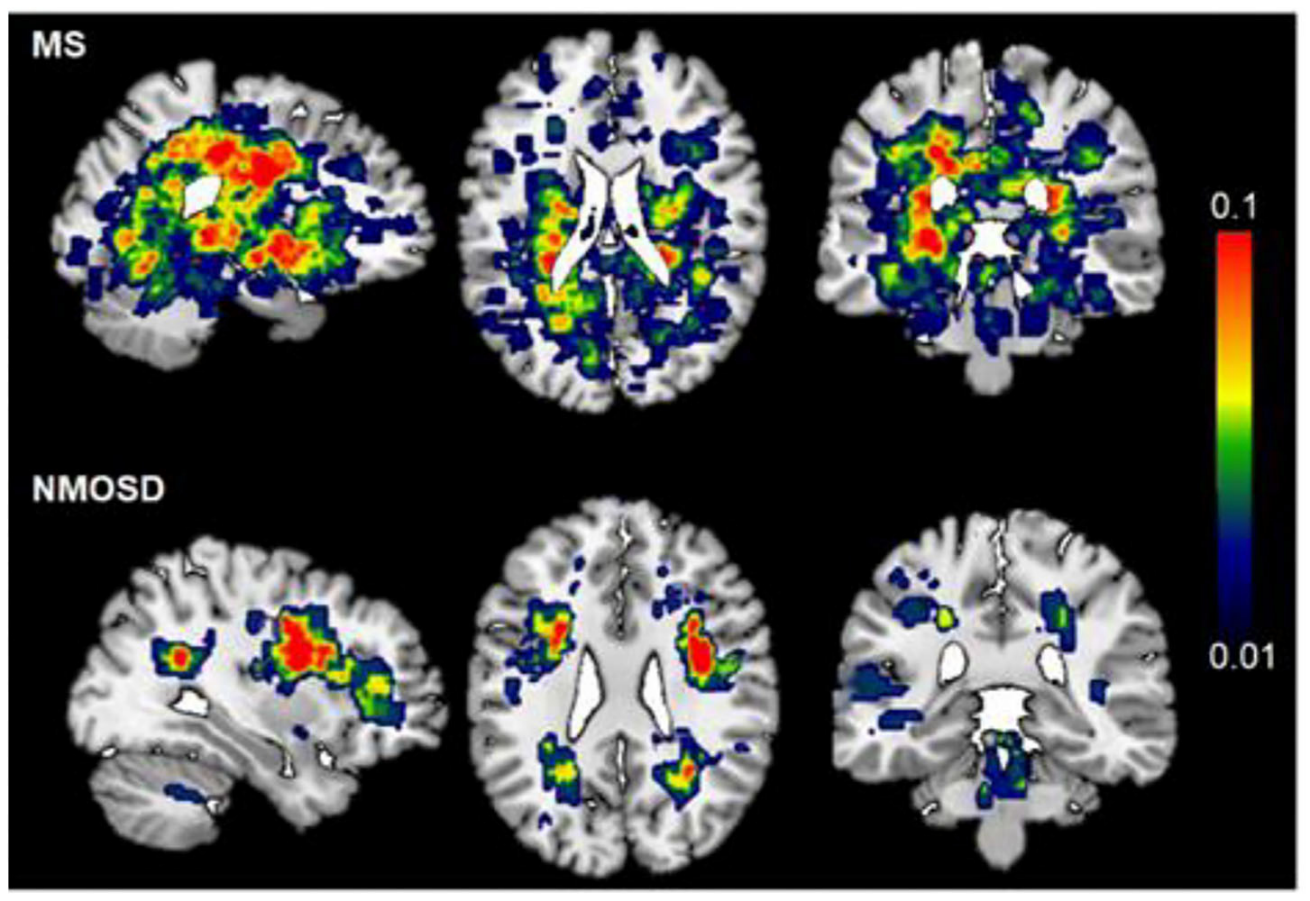
Probability map of brain lesions in patients with NMOSD and MS. T2-weighted lesion probability maps in MS (upper, *n* = 85) and NMOSD (lower panel, *n* = 90) patients. The lesion mask of each patient was created on top of the Montreal Neurological Institute standard 2 mm T1WI template brain (MNI152). The color bar denotes the probability range (1–10%). MS has more brain lesions than NMOSD. The highest probability of brain lesion distribution was periventricular in MS patients, deep white matter in NMOSD.

Both NMOSD and MS lesions are scattered in the supratentorial and subtentorial parenchyma, and mainly distributed in the deep white matter area of the brain (Figure 1). Compared with NMOSD, MS lesions involve a wider range of the brain, and are mainly located in periventricular regions and deep white matter. The highest probability distribution for NMOSD brain lesions was in the deep white matter area of the brain, whereas MS lesion is located around the bilateral ventricle. Figure 1 shows the probability map of brain lesions in patients with NMOSD and MS.

### Different Patterns of Ventricle Enlargement in MS and NMOSD

**Figure 2:**
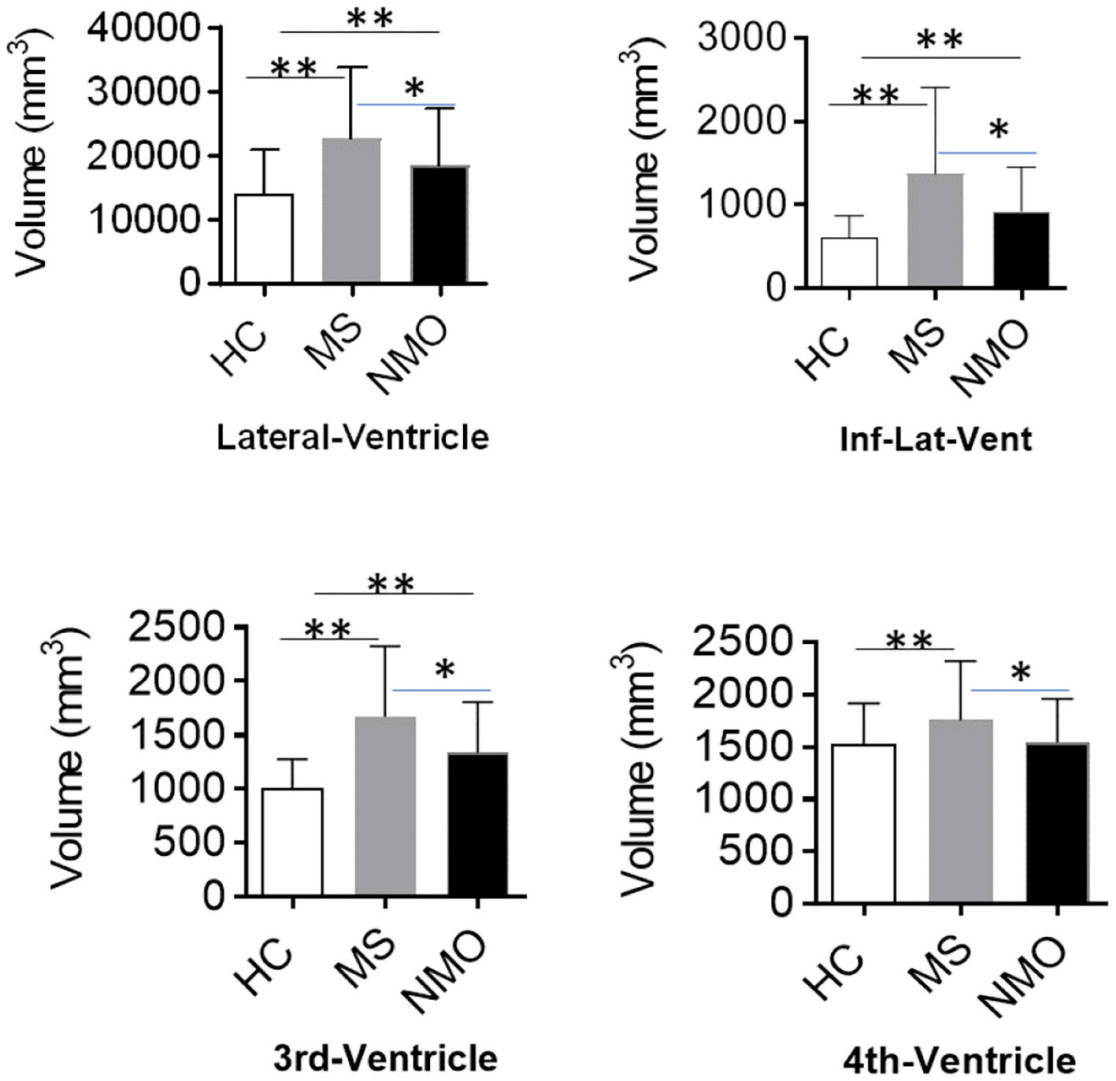
Different patterns of ventricle enlargement in MS and NMOSD. The ventricular volume, including lateral, 3rd, and 4th ventricles, showed a remarkable difference among HCs, MS, and NMOSD patients. Patients with MS exhibited ubiquitous ventricle enlargement, whereas NMOSD ventricle enlargement is mainly in the lateral and 3rd ventricles. The 4th ventricle volume decreased in MS rather with NMOSD. Inf-Lat-Vent, temporal horn of the lateral ventricle. *p < 0.05, **p < 0.001.

Compared with HCs, both NMOSD and MS showed an enlarged lateral ventricle (*p* < 0.001). The temporal horn of the lateral ventricle (inferior lateral ventricle) also expanded accordingly (HC vs. MS, *p* < 0.001; HC vs. NMOSD, *p* < 0.001). These changes are mirrored in the third ventricle (*p* < 0.001). However, the fourth ventricle was enlarged in MS patients, but no differences were shown in NMOSD patients compared with healthy controls (HC vs. MS, *p* < 0.001; HC vs. NMOSD, *p* = 0.321). Compared with NMOSD, the enlargement of the lateral ventricle is more pronounced in MS (*p* = 0.045); correspondingly, the temporal horn of the lateral ventricle (Inf-Lat-Vent) exhibits the same trend (*p* = 0.009). The third and fourth ventricles are dramatically larger in MS than NMOSD (*p* = 0.003, *p* = 0.007). These changes indicate that MS patients undergo widely extensive expansion of the ventricle (Figure 2). Correspondingly, the expansion of the ventricle in NMOSD patients was concentrated in the lateral and third ventricles. Ventricle expansion is more pronounced in MS patients, and NMO patients had an expansion of the ventricle between healthy controls and MS.

### Brain Atrophy in MS and NMOSD

Calculated thickness, volume, and surface area of the cortex reflect the gray area changes. MS had lower total GM volume (601.7 ± 60.7 vs. 627.5 ± 53.9 cm^3^) and cortex volume (447.9 ± 47.3 vs. 464.2 ± 43.6 cm^3^) and lower WM volumes (415.9 ± 59.7 vs. 452.8 ± 40.6 cm^3^) and higher ventricular (lateral, third, and fourth ventricles) volumes than HCs. NMOSD patients also experienced a decrease in total GM volume (591.6 ± 47.6 cm^3^), cortex volume (437.6 ± 38.1 cm^3^), and WM volumes (429.7 ± 44.7 cm^3^) compared with HCs. However, when compared with MS, only subcortical gray volume showed statistical differences (53.76 ± 4.84 vs. 50.85 ± 7.14 cm^3^, *p* < 0.05).

**Figure 3:**
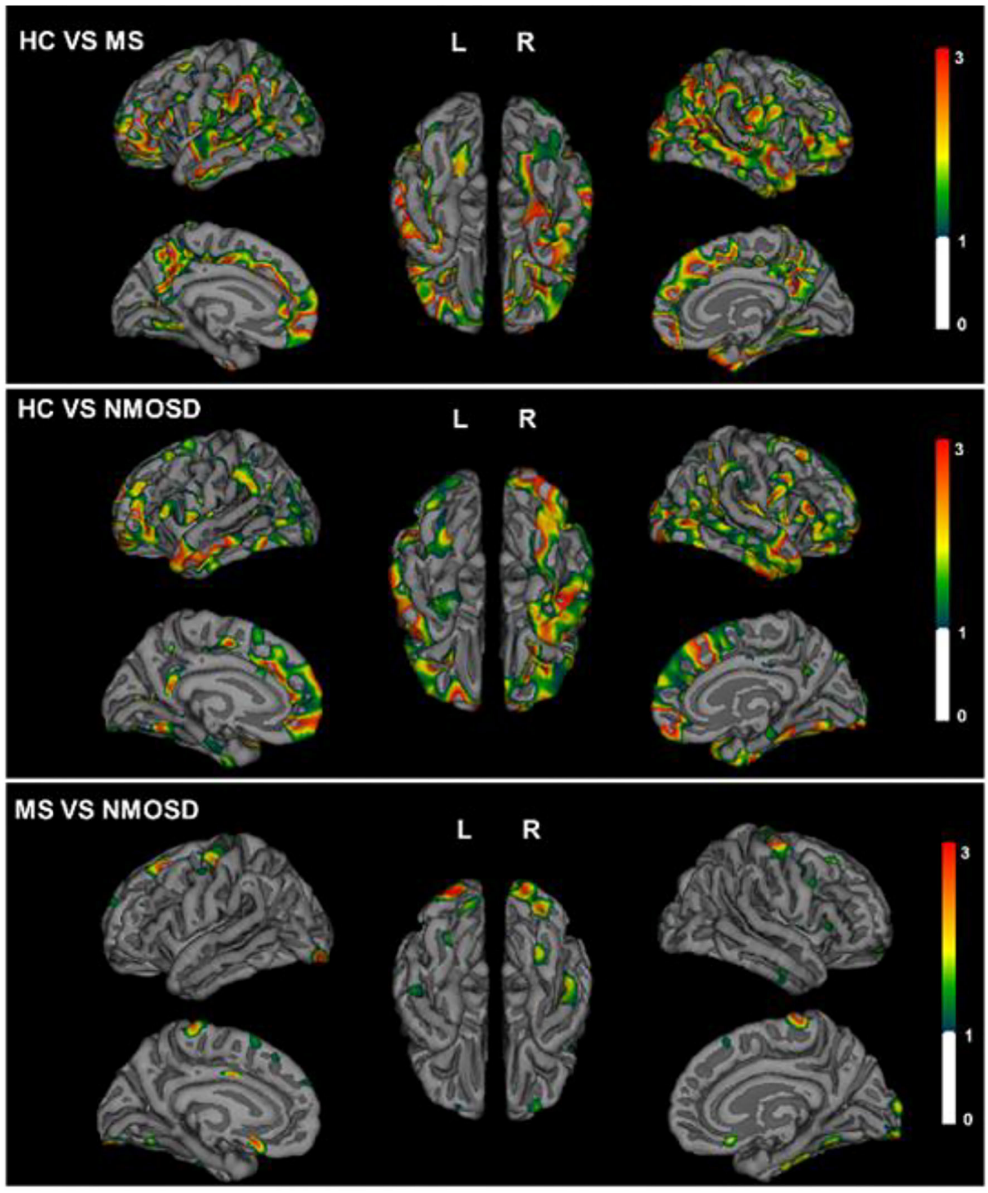
Topographical distribution of group differences in cortical thickness for MS and NMOSD. The Statistical *p* maps showed global decreased thickness of cortex in MS (top) and NMOSD (middle) compared with HCs. Difference of pre-cuneus, parahippocampal, and lateral occipital lobe between MS and NMOSD (bottom). The color bar represents corrected –log 10(*p*-value) range from 0 to 3.

In patients with MS, a significant reduction in the thickness of the cortex is shown compared with healthy controls, especially in lateral orbitofrontal, post-central, and middle temporal lobe of the left hemisphere and parahippocampal, rostral middle frontal, superior frontal, and pre-cuneus of the right hemisphere. In NMOSD patients, the decrease in the thickness of the cortex was mainly located in both superior temporal and rostral middle frontal, left inferior temporal lobe, right superior frontal, parstriangularis, and inferior parietal lobe. However, when MS and NMOSD were compared, pronounced cortical atrophy was only significant in pre-cuneus, parahippocampal, and lateral occipital lobe. In the areas where NMOSD and healthy controls showed a decrease in the thickness of the cortex, there was no statistical difference after comparisons with MS. In other words, MS and NMOSD show the same degree of brain atrophy in these areas. It is worth noting that NMOSD patients have fewer brain lesions than patients with MS, especially those adjacent to the lateral ventricle. Topographic distribution of significant group differences in thickness of cortex is displayed in Figure 3.

**Figure 4:**
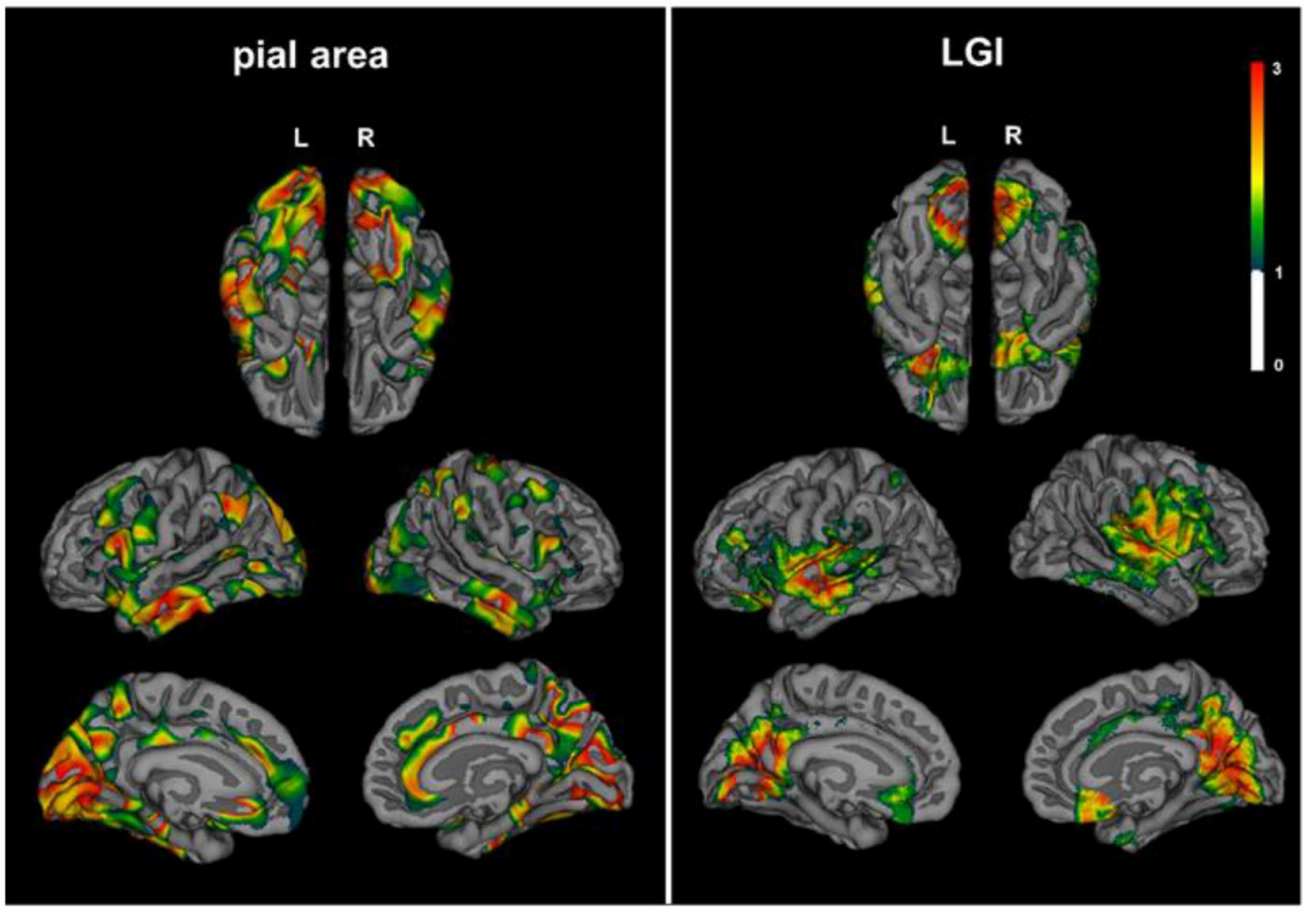
Decreased cortical pial surface area and LGI in NMOSD. Patients with NMOSD exhibited decreased pial surface area (left) and LGI (right) compared with HCs. Topographical distribution map of pial surface area showed group differences in lingual of left hemisphere, pre-cuneus, lateral occipital, and middle temporal lobe of the right hemisphere. LGI descended in lingual and orbitofrontal of the left hemisphere and supramarginal, pre-cuneus, and orbitofrontal lobe of the right hemisphere. The color bar represents corrected –log 10(*p*-value) that ranged from 0 to 3. LGI, local gyrification index.

Patients with NMOSD exhibited decreased pial surface area in lingual of left hemisphere, and pre-cuneus, lateral occipital, and middle temporal lobe of right hemisphere compared with HCs. LGI, which quantified the amount of cortex buried within the sulcal folds, also descended in lingual and orbitofrontal of left hemisphere and supramarginal, pre-cuneus, and orbitofrontal lobe of right hemisphere (Figure 4).

### Cortical Gray Atrophy Correlating With Age and Disease History

Patients with NMOSD experienced extensive widely cortex atrophy. Covariates including age, AQP-4 status, history, annualized relapse rate, the length of myelitis, periventricular lesion, and brain lesion volume all entered the linear regression model. Linear regression analysis revealed that cortex atrophy, as measured by total gray volume and cortex volume, was predicted by increased age (standardized β = −0.404, *p* = 0.001) and longer disease duration (standardized β = −0.311, *p* = 0.006). Only the disease duration was associated with subcortical gray atrophy (standardized β = −0.303, *p* = 0.017). This demonstrates that cortex atrophy is an independent process related to the disease duration in patients with NMOSD (Table 2).

**Table 2:** Linear regression of cortical gray volume in patients with NMOSD.

	**Total gray volume**			**Cortex volume**			**Subcortical gray volume**		
	**Unstandardized coefficients**		***P*-value**	**Unstandardized coefficients**		***P*-value**	**Unstandardized coefficients**		***P*-value**
	***B***	**Std. Error**		***B***	**Std. Error**		***B***	**Std. Error**	
Age	−1323.223	372.879	0.001	−1003.172	303.463	0.001	−27.883	41.850	0.507
AQP-4 Ab	−2917.013	10662.025	0.785	719.389	8677.151	0.934	−585.625	1196.642	0.626
Disease duration	−3963.141	1384.280	0.006	−3223.617	1126.578	0.006	−381.029	155.363	0.017
ARR	−1285.521	3929.718	0.745	−1674.297	3193.488	0.602	−405.014	437.389	0.385
Periventricular lesion	−10308.128	11287.389	0.364	−5723.828	9186.095	0.535	−1589.491	1266.829	0.214
Brain lesion volume (mm^3^)	813.260	1821.211	0.657	535.879	1482.169	0.719	−55.723	204.402	0.786
Spinal lesion length	346.279	1221.607	0.778	178.011	994.189	0.858	105.646	137.106	0.444

*All predictors entered. AQP-4 Ab: 0 = negative, 1 = positive. ARR, annualized relapse rate. Periventricular lesion: 0 = No, 1 = Yes. Spinal lesion length: number of vertebrae corresponding to lesions*.

## Discussion

In the present study, patients with MS demonstrated widely extensive ventricle enlargement. NMOSD patients mainly experienced ventricular enlargement in the lateral ventricle and third ventricle. The degree of ventricle enlargement in NMOSD were lower than those with MS. Both MS and NMOSD exhibited global brain atrophy compared with HCs. Notably, thickness of the cortex were only different in the pre-cuneus, parahippocampal, and lateral occipital lobe between NMOSD and MS. NMOSD patients exhibited decreased pial surface area and LGI compared with HCs. Cortex atrophy of NMOSD may be associated with age and disease duration rather than brain and spinal lesions.

Distinct patterns of ventricle enlargement in MS and NMOSD were confirmed in our study. MS patients showed widespread ventricle enlargement, whereas changes in NMOSD were less pronounced and localized mainly to the lateral and third ventricles. The distribution of brain lesions for both diseases are clearly different (Figure 1), yet whether in MS or NMOSD, the distribution of lesions is closely related to the ventricular system. Brain lesions are more likely adjacent to the body of the lateral ventricle in MS than NMOSD, suggesting that the mechanism of the enlargement may be different between MS and NMOSD. Larger lateral and third ventricle volumes in NMOSD may not be attributed to limited adjacent lesions. Lesions along the ependyma, where the basolateral membrane extensively express AQP-4, is distinctive in NMOSD (23, 24). Ventriculitis and leptomeningitis were confirmed with NMO IgG-induced pathological alterations, which extended beyond perivascular astrocytic foot processes to include the pia, ependyma, and choroid plexus. AQP-4 immunoreactivity was lost from intact ependymocytes into the subependymal parenchyma accompanied by eosinophilic and granulocyte infiltration (12). Moreover, obstructive hydrocephalus was recently observed in patients with NMOSD (14).

Our results suggest that cortical atrophy in NMOSD was global when compared with HCs, but significant differences were found in parieto-occipital lobe and parahippocampus between NMOSD and MS. It is well-known that inflammation is not the sole driver of accumulating disability, as neurodegeneration is widespread from the early disease course in MS (25). Notably, patients with NMOSD showed the same degree of brain atrophy as MS in our study; however, there are far fewer brain lesions in NMOSD. The significant global thinning of the cerebral cortex is consistent with previous findings in 91 patients with NMOSD (26). In this study, more widespread cortical thinning was confirmed in both MS and NMOSD patients. This is also in line with our previous results, where NMOSD had a lower normalized GM volume than HCs. However, several small-sample studies exhibited limited regional brain atrophy, mainly in the visual cortex (15, 17). On the one hand, surface-based morphometry provides distinct surface area complexity rather than voxel-based morphometry using volumetric data alone. On the other hand, previous imaging studies have highlighted differences between NMO and MS, ignoring the changes that may exist together. Positive predictive value of all gray matter measures together is 89% in NMOSD vs. HCs, but only 73% in NMOSD vs. MS (27). The volumes of the thalamus were the most important deep gray matter structure to distinguish MS from NMOSD. In other words, there is no difference in most of the cortical volume in NMOSD and MS.

The decreased cortex volume was associated with age and disease duration rather than annualized relapse rate and myelitis lesions in NMOSD. Therefore, the mechanism of axonal degeneration secondary to damage of the spinal cord and optic nerves cannot fully explain the extensive reductions in the gray matter volume, which also does not directly correlate with the number of white matter lesions. Degeneration is a partially independent pathological process in MS. However, progressive disease stage is rarely seen in NMO, and neurodegeneration in NMOSD may be completely dependent on inflammatory relapses (2). Leptomeningeal inflammation drives the underlying change with varying degrees of severity and pathogenetic mechanisms in MS and NMOSD. The cortical lesions are characterized by demyelination, subtle neuroaxonal degeneration, and ectopic B-cell follicle-like structures in MS meninges (28). In contrast, meningeal inflammation was abundant, but B-cell follicle-like structures were not detected in NMOSD. In addition, in NMOSD, non-lytic reaction of AQP-4 negative astrocytes were found in cortical layer I and abundant reactive astrocytes with preservation of AQP-4 sensitivity in cortical layers II–VI (12, 13). Taken together, these data suggest that astrocytopathy targeted sub-pial AQP-4 have profound pathogenic and neurologic consequences.

The pathological changes in cortical gray matter and ependyma in NMOSD were confirmed by our MRI findings. Histopathology of NMO patients suggested ventriculitis and leptomeningitis may occur at the pial glia limitans, the ependymal surface, and the choroid plexus. In our study, both the expansion of the ventricle and the reduction of the thickness of the cortex, respectively, co-exist. Surface area and LGI presented a reduction in NMOSD compared with HCs. Diffuse gray matter damage is probably driven by NMO IgG-induced pathological mechanism. The interfaces between blood and CSF and between CSF and brain are key sites for NMO IgG entrance. AQP-4 is also expressed at the pial glia limitans and the ependyma of CSF–brain interfaces. NMO IgG and soluble inflammatory factors may be accessible to AQP-4 through the blood–CSF barrier, then circulates through the ventricles and over the surface of the brain.

A limitation of this study is based on a cross-sectional design rather than a longitudinal cohort. A longitudinal study would provide in-depth insight into annual changes of cortical thickness. However, the large cohort of 90 NMOSD patients ensures that robust results can be obtained in the present study. In addition, the link between atrophy of specific brain regions on MRI and the associated cognitive domains is not explored. Unfortunately, comprehensive cognitive testing has not been obtained in the present cohort. Furthermore, certain disease-modifying drugs (DMDs) may slow the progression of brain atrophy in MS. However, it was not until last year that betaseron and teriflunomide were approved for the Chinese market; most of our patients do not use disease-modifying therapy drugs.

In conclusion, MS showed widespread expansion of all ventricles, whereas NMOSD mainly displayed enlargement of the lateral ventricle and the third ventricle. The extent of ventricular expansion in NMOSD is between MS and HC. NMOSD patients demonstrated global brain atrophy similar to MS, but the thinning of cortex is not associated with brain and spinal lesions. The decreased LGI and the enlarged ventricles also indicate that the CSF–brain interfaces may be a core component in the AQP-4 evoked pathological changes.

## Data Availability Statement

The raw data supporting the conclusions of this article will be made available by the authors, without undue reservation.

## Ethics Statement

The studies involving human participants were reviewed and approved by Tianjin Medical University General Hospital and Beijing Tiantan Hospital Ethics Committee. The patients/participants provided their written informed consent to participate in this study.

## Author Contributions

F-DS and XZ formulated the conception and design of this study. MF, TL, LS, and YM contributed to the acquisition and analysis of data, and to critical revisions of the article. F-DS and D-CT funding support. F-DS, XZ, D-CT, YL, and XW drafted the article and prepared the figures. All authors contributed to the article and approved the submitted version.

## Conflict of Interest

The authors declare that the research was conducted in the absence of any commercial or financial relationships that could be construed as a potential conflict of interest.
