# Supplementary material for: Cortical Thinning and Ventricle Enlargement in Neuromyelitis Optica Spectrum Disorders
Source: Front Neurol. 2020 Aug 27;11:872. doi: 10.3389/fneur.2020.00872 (PMC7481470; doi:10.3389/fneur.2020.00872)
Supplement: Supplementary file 1 [file Table_1.DOCX]

| **Supplementary Table 1 MRI measures in AQP-4 positive and negative NMOSD patients** | | |
| --- | --- | --- |
| variable | NMOSD with AQP-4 positive  (n=59) | NMOSD with AQP-4 negative  (n=31) |
| **Brain segment volume** |  |  |
| Total Gray volume(cm^3^), (MD ± SD) | 586.4±45.9 | 602.1±50.2 |
| Cortex volume(cm^3^), (MD ± SD) | 434.2±37.5 | 444.7±39.2 |
| Sub Cortex Gray volume(cm^3^), (MD ± SD) | 53.60±5.12 | 54.08±4.30 |
| Cerebral White Matter volume(cm^3^), (MD ± SD) | 426.9±39.2 | 435.4±54.7 |
| **Ventricle volume** |  |  |
| Lateral Ventricle volume(cm^3^), (MD ± SD) | 18.1±8.8 | 18.1±5.7 |
| Inf-Lat-Vent volume (cm^3^), (MD ± SD) | 0.97±0.6 | 0.93±0.4 |
| 3rd-Ventricle volume(cm^3^), (MD ± SD) | 1.27±0.4 | 1.38±0.4 |
| 4th-Ventricle volume(cm^3^), (MD ± SD) | 1.51±0.4 | 1.57±0.4 |
| NMOSD=neuromyelitis optica spectrum disorder; Inf-Lat-Vent= temporal horn of the lateral ventricle; MD=median; SD=standard deviation; *P<0.05. | | |
